# An improved fruit transcriptome and the identification of the candidate genes involved in fruit abscission induced by carbohydrate stress in litchi

**DOI:** 10.3389/fpls.2015.00439

**Published:** 2015-06-15

**Authors:** Caiqin Li, Yan Wang, Xuming Huang, Jiang Li, Huicong Wang, Jianguo Li

**Affiliations:** ^1^State Key Laboratory for Conservation and Utilization of Subtropical Agro-Bioresources, China Litchi Research Center, South China Agricultural University, GuangzhouChina; ^2^Physiological Laboratory for South China Fruits, College of Horticulture, South China Agricultural University, GuangzhouChina; ^3^Beijing Genomics Institute at Shenzhen, ShenzhenChina

**Keywords:** *Litchi chinensis* Sonn., transcriptome, carbohydrate stress, fruit abscission, *de novo* assembly, digital transcript abundance, genes

## Abstract

Massive young fruit abscission usually causes low and unstable yield in litchi (*Litchi chinensis* Sonn.), an important fruit crop cultivated in tropical and subtropical areas. However, the molecular mechanism of fruit drop has not been fully characterized. This study aimed at identification of molecular components involved in fruitlet abscission in litchi, for which reference genome is not available at present. An improved *de novo* transcriptome assembly was firstly achieved by using an optimized assembly software, Trinity. Using improved transcriptome assembly as reference, digital transcript abundance (DTA) profiling was performed to screen and identify candidate genes involved in fruit abscission induced by girdling plus defoliation (GPD), a treatment significantly decreased the soluble sugar contents causing carbohydrate stress to fruit. Our results showed that the increasing fruit abscission rate after GPD treatment was associated with higher ethylene production and lower glucose levels in fruit. A total of 2,771 differentially expressed genes were identified as GPD-responsive genes, 857 of which were defined by GO and KEGG enrichment analyses as the candidate genes involved in fruit abscission process. These genes were involved in diverse metabolic processes and pathways, including carbohydrate metabolism, plant hormone synthesis, and signaling, transcription factor activity and cell wall modification that were rapidly induced in the early stages (within 2 days after treatment). qRT-PCR was used to explore the expression pattern of 15 selected candidate genes in the abscission zone, pericarp, and seed, which confirmed the accuracy of our DTA data. More detailed information for different functional categories was also analyzed. This study profiled the gene expression related to fruit abscission induced by carbohydrate stress at whole transcriptome level and thus provided a better understanding of the regulatory mechanism of young fruit abscission in litchi.

## Introduction

Litchi (*Litchi chinensis* Sonn.) is one of the important economic fruit crops widely grown in south China and Southeast Asian areas. The excessive fruit drop during fruit development is a major problem causing serious economic loss for the growers. Yuan and Huang (1988) reported three to four waves of fruit drop throughout fruit development in different cultivars based on relative abscission rate. Wave I, wave II, and wave III of abscission occurred around 1, 3, and 6–7 weeks after full bloom, respectively, but wave IV was specific to cultivars with aborted seeds and occurred 2–3 weeks before harvest. Overall, less than 5% of the initial female flowers can develop into mature fruit (Stern et al., 1993, 1995; Mitra et al., 2003). Endogenous hormones (Yuan and Huang, 1988; Xiang et al., 1995; Li et al., 2004) and carbohydrates (Yuan and Huang, 1992, 1993; Zhou et al., 1999; Hieke et al., 2002; Chang and Lin, 2007, 2008; Yuan et al., 2009) are proposed to play key roles in the regulation of fruit abscission in litchi. In citrus, it has been suggested that the subsequent fruit development is mostly supported by nutrient supply after hormonal activation of initial fruit growth (Talon et al., 1997). Thus, once mineral and water requirements are satisfied, competition for photo-assimilates is thought to be responsible for fruit drop (Moss et al., 1972; Powell and Krezdorn, 1977; Gómez-Cadenas et al., 2000). Defoliation experiments showed that the sucrose status of the fruitlet is a major factor in the regulatory mechanism of fruit abscission in citrus (Mehouachi et al., 1995; Gómez-Cadenas et al., 2000) and apple (Berüter and Droz, 1991). On girdled macadamia branches, the number of fruit retained was dependent on the number of available leaves, which determined the availability of assimilates to the fruit (Trueman and Turnbull, 1994). When applying pedicel girdling at the proximal portion to induce citrus fruitlet drop, reduction of soluble sugars accumulation and induction of ethylene production occurred as well (Iglesias et al., 2006). In litchi, Yuan and Huang (1988) demonstrated that litchi fruit set was strongly dependent on current photosynthesis. Treatment of girdling (a ring of bark and cambium was removed from the branch base) plus defoliation (100% leaf removal) in litchi reduced endogenous IAA concentration and increased the transcript level of two IAA-responsive genes (*LcAUX/IAA1* and *LcSAUR1*), one cell wall degrading enzyme gene (*LcPG1*) and one ethylene biosynthetic gene (*Lc-ACO1*), in contrast to the decreasing accumulation of auxin response factor (*LcARF1*) mRNA, with the concomitant increase in fruit drop (Kuang et al., 2012; Peng et al., 2013; Wu et al., 2013). However, a deep knowledge of the molecular events involved in fruit abscission induced by carbohydrate stress is still missing.

With the development of microarray and next generation sequencing technology, global transcriptome analyses have been widely used to investigate the molecular regulatory networks on various organs abscission, such as flower (Cho et al., 2008; Meir et al., 2010; Wang et al., 2013), leaves (Agustí et al., 2009), shoot tips (Zhang et al., 2014), young fruit (Botton et al., 2011; Zhu et al., 2011), and mature fruit (Corbacho et al., 2013; Gil-Amado and Gomez-Jimenez, 2013). In litchi, the reference genome is not available at present. We previously assembled the first fruit transcriptome by SOAPdenovo software and discovered 1,039 differentially regulated unigenes responding to shading-induced fruitlet abscission (Li et al., 2013). However, other assembly software, such as Trinity and Oases, has been shown to obtain superior overall results compared to SOAPdenovo (Zhao et al., 2011; Ghangal et al., 2013). Here, we firstly used four publicly available assemblers to assess the litchi fruit RNA-Seq data generated previously (Li et al., 2013). And then, we compared GPD-induced fruit drop with the CK via gene expression profiling performed on the three pooled tissues (fruit AZ, pericarp and seed), sampled 2, 4, and 7 days after GPD treatment. A number of pathways and candidate genes associated with fruit abscission were screened and identified. Our results provided more clues for a better understanding of the mechanisms of fruit abscission induced by carbohydrate stress in litchi.

## Materials and Methods

### Plant Materials and Treatments

Three randomly selected 9-years-old litchi trees (*Litchi chinensis* Sonn. cv. Wuye) grown in an orchard in South China Agricultural University (Guangzhou, China) were chosen. Twenty fruit-bearing shoots with similar diameter located in different directions from each tree were tagged. Ten of them were treated with girdling (a ring of bark about 0.5 cm in width and cambium was removed from the branch base) followed by defoliation (removing all leaves above the girdle) at 35 days after anthesis (GPD treatment), while the remaining untreated shoots were used as CK. Three out of ten treated shoots were used to monitor fruit abscission dynamic and the others were used for sampling. Samples were collected at 0, 2, 4, and 7 days after treatment. Fruit were dissected using a sharp razor blade into pericarp and seed, while AZ was excised by cutting around 2 mm at each side of the abscission fracture plane. After separation, all tissues were quickly frozen in liquid nitrogen and stored at -80°C for future analysis. Each tree was treated as a biological replicate.

### Determination of Fruit Abscission and Ethylene Production Rate of Fruit

Cumulative fruit abscission rate and relative fruit abscission rate were calculated according to our previous method (Kuang et al., 2012). Ethylene production was measured according to the method described by Yan et al. (2011) with some modifications. Two fruit from each treatment on each tree were collected and enclosed in a 25 mL airtight syringe equipped with a rubber piston for 2 h at 25°C. Air within the syringe was forced into an airtight container filled with saturated salt water with a needle inserted to allow replacement. After all the air samples were collected in the experiment, 1 mL air sample was then withdrawn from the headspace of the container with a syringe and injected into a GC-17A gas chromatograph (Shimadzu, Kyoto, Japan) fitted with a flame ionization detector and an activated alumina column (200 cm × 0.3 cm). The injector temperature was 120°C; the column temperature was kept at 60°C and the detector temperature at 60°C. Helium was used as carrier gas at a flow rate of 30 mL⋅min^-1^. The ethylene production rate was expressed as microliters of C_2_H_4_ kg^-1^⋅h^-1^.

### Extraction and Measurement of Sugars

Sugars were extracted and determined according to the protocol of Hu et al. (2005) with modifications. Pericarp or seed samples (200 mg) were ground in liquid nitrogen with a mortar and then extracted with 5 mL of 80% ethanol (v/v) at 80°C for 40 min. The extracts were then centrifuged at 12,000 *g* for 10 min at room temperature. The ethanol extraction step was repeated once. The supernatant resulting from the two extractions was combined and diluted with 80% ethanol to 10 mL. An aliquot 2 mL of the supernatant was dried with a rotary evaporator for 8 h and then dissolved in 2 mL of distilled water. After dissolution, the supernatant (1 mL) was passed through a Sep-Pak^®^1cc (100 mg) C18 cartridge (Waters Corporation, Milford, MA, USA). Sugars were measured by high-performance liquid chromatography (HPLC) using an Agilent 1200 HPLC system (Agilent Technologies, Waldbronn, Germany) with a G1362A refractive index detector cell maintained at 40°C. A Transgenomic CARB Sep Coregel 87°C column (CHO-99-5860) with a guard column cartridge (Transgenomic CARB Sep Coregel 87°C cartridge) was used. The column was maintained at 80°C with a thermostatic column compartment. The injection volume was 10 μL. Samples were eluted with double-distilled water. The flow rate was 0.6 mL⋅min^-1^. The quantification of sugars was performed according to external standard solution calibration (sugar standards were purchased from Sigma Chemical Co.).

### Comparison of Litchi Fruit Transcriptome Assembly

In this study, we compared the performance of four commonly used *de novo* short read assembly softwares including SOAPdenovo^1^ (version 1.04, Li et al., 2009), SOAPdenovo-Trans^2^ (version 1.03, Xie et al., 2014), Velvet-Oases^3^ (Velvet: version 1.2.09, and Oases^4^: version 0.2.08, Schulz et al., 2012) and Trinity^5^ (version 2013-02-25, Grabherr et al., 2011). These programs have been developed for short reads assembly using a de Bruijn graph algorithm (Zerbino and Birney, 2008). SOAPdenovo-Trans was used to assemble *k-mer* lengths of 23–41 with a step length of 4, Velvet-Oases were used to assemble *k-mer* lengths of 19–43 with a step length of 6, SOAPdenovo and Trinity was used to assemble a single *k-mer* length of 25. Any redundant fragments generated from four softwares were removed by TGICL (version 2.1) and Phrap (version release 23.0) assembler to get final genes. Following parameters were used to ensure a high quality of assembly: a minimum of 95% identity, a minimum of 35 overlapping bases, a minimum of 35 scores and a maximum of 25 unmatched overhanging bases at sequence ends. Based on sequence similarity, the genes were divided into two classes: clusters (prefixed with ‘CL’) and singletons (prefixed with ‘unigene’). In a cluster, the mutual similarity was more than 70%. Clean reads were mapped back onto respective assembled genes using SOAPaligner (version: 2.21) to check the integrity of assembled results. Total number of genes, N50 and average gene length were also taken into consideration to evaluate the quality of transcriptome assemblies. The best transcriptome assembly was used for following analyses.

Functional annotation of the assembled genes was predicted based on the highest similarity in the following databases: Nr^6^ (NCBI non-redundant protein sequences), Ntveer6 (NCBI non-redundant nucleotide sequences), COGveer6 (Clusters of Orthologous Groups of proteins, COG), Swiss-Prot^7^ (A manually annotated and reviewed protein sequence database), KO^8^ (KEGG Orthology database) and GO. GO functional annotation was performed by Blast2GO (v2.5.0) software (Conesa et al., 2005). The raw sequence data used in this study has been submitted to National Center for Biotechnology Information (NCBI) Short Read Archive (SRA) with accession number SRX255051 (Li et al., 2013). The sequences assembled by Trinity have been stored in NCBI’s Transcriptome Shotgun Assembly (TSA) database which can be accessed using the Gene-IDs listed in Supplementary Table S1.

### Digital Transcript Abundance Library Preparation and Illumina Sequencing

Total RNA from three tissues (AZ, pericarp, and seed) was isolated using Column Plant RNAout 2.0 kit (TIANDZ, Inc., China). The quantity and quality of RNA samples were evaluated using 2100 Bioanalyzer (Agilent Technologies, Santa Clara, CA, USA). Equal total RNA from different tissues sampled at specific time points of the same treatment were pooled to construct six libraries named CK2, CK4, CK7, GPD2, GPD4, and GPD7. For example, CK2 and GPD2 were the libraries from tissues harvested at 2 days in the CK and the GPD treatment, respectively. After RNA extraction, mRNA purification by Oligo (dT), fragmentation, cDNA synthesis by random hexamer primers, size selection and PCR amplification were sequentially performed by BGI-Shenzhen as described previously (Li et al., 2013). DTA datasets were deposited in the NCBI’s SRA database with the accession numbers as follows: SRX847812 (CK2), SRX847822 (CK4), SRX847823 (CK7), SRX847824 (GPD2), SRX847825 (GPD4), and SRX847826 (GPD7).

### Data Analysis for Digital Transcript Abundance Profiles

High-quality reads filtered through the standard Illumina pipeline to remove the low-quality reads and those containing adaptor/primer contaminations were used for further downstream processing. All clean reads were mapped back to the updated transcriptome reference database using SOAPaligner (version 2.21) allowing up to two nucleotide mismatches with the parameters of “-m 0 -x 1000 -s 28 -l 32 -v 2 -r 2,” which are specified on http://soap.genomics.org.cn/soapaligner.html. For gene expression analysis, the number of unambiguous clean reads for each gene was calculated and normalized to RPKM (Reads Per Kilobase per Million reads; Mortazavi et al., 2008). Three paired-libraries including CK2 vs. GPD2, CK4 vs. GPD4, and CK7 vs. GPD7 were used to analyze the differential gene expression, according to the method described in Audic and Claverie (1997). Two filter criteria were used to identify DEGs: a fold change in transcript levels ≥4 and a FDR (False Discovery Rate) value ≤0.001. For functional and pathway-enrichment analyses, all DEGs were mapped to terms in GO and KEGG databases. Heatmaps showing expression profiles (log_2_ fold change) were generated using the MultiExperiment Viewer (MeV, v4.9). Hierarchical clustering was performed by Euclidean distance matrix with a complete linkage rule using MeV. DEGs significantly enriched in GO term analysis (FDR ≤ 0.05) or enriched in KEGG pathway (*Q* value ≤ 0.05) were screened to be the candidate genes involved in the fruit abscission process.

### Quantitative Real-Time PCR Analysis

To validate the accuracy of our DTA profiling results, 15 randomly selected candidate genes were evaluated by quantitative real-time PCR (qRT-PCR) after GPD-treatment in AZ, pericarp, and seed of litchi. The list of gene-specific primers was shown in Supplementary Table S2. RNA reverse transcription, qRT-PCR reaction, data normalization, and calculation were performed as described previously (Li et al., 2013). Values of each time point were means of three biological replicates.

## Results

### Transcriptome Assembly Optimization

Here, four publicly available assemblers were used to assess the litchi fruit RNA-Seq data generated previously (Li et al., 2013). For SOAPdenovo-Trans, increased *k-mer* value (ranging from 23 to 41) led to the quality deterioration in the generated assembly with the reduction of average and N50 gene lengths, so the 23-*mer* in SOAPdenovo-Trans was selected for final assembly. In contrast, assembly generated by Velvet-Oases at *k* = 31 was found to obtain better quality when different *k-mer* lengths ranging from 19 to 43 (Supplementary Figure S1) were tested. Compared to other three assembly software, Trinity generated the best assembly with the largest average (776 bp) and N50 (1,198 bp) gene lengths and utilized 88.70% of the total reads, SOAPdenovo-Trans (*k* = 23) came in the second, and SOAPdenovo used in our previous study was the worst in the category due to the lowest utilized reads (**Table 1**). Moreover, the assembly programs varied widely in the lengths distributions of the genes. Trinity produced superior results in much larger numbers of long-genes (>1 kb) and fewer short-transcripts (200–500 bp; Supplementary Figure S2A). Thus, the transcriptome assembled by Trinity was used for functional annotation. This improved assembly had 45,370 non-redundant genes without ‘N,’ comprising a total length of 35,197,676 bp, with 22,706 genes (50.05%) longer than 500 bp, 11,932 genes (26.30%) longer than 1000 bp, and 2,918 (6.43%) genes longer than 2000 bp (Supplementary Figure S2A). To evaluate the accuracy of the assembled sequence, all the usable reads were re-aligned onto the genes (Supplementary Figure S2B), showing that there were 42,661 genes (94.03%) with very high reads coverage (90 ∼ 100%).

**Table 1:** Comparison of different assembled softwares.

	SOAPdenovo	SOAPdenovo- Trans (*k* = 23)	Velvetoases (*k* = 31)	Trinity
Number of genes	57,050	32,455	59,461	45,370
Maximum length (bp)	10,687	8,220	4,979	9,098
Average length (bp)	601	679	442	776
GC (%)	40.88	43.09	42.57	42.42
N (%)	1.37	0.07	0.50	0
N50 length (bp)	811	997	491	1,198
Reads utilization (%)	39.32	73.26	60.69	88.70

### Changes in Fruit Abscission Rate, Ethylene Production, and Sugars Contents in Response to GPD Treatment

**FIGURE 1:**
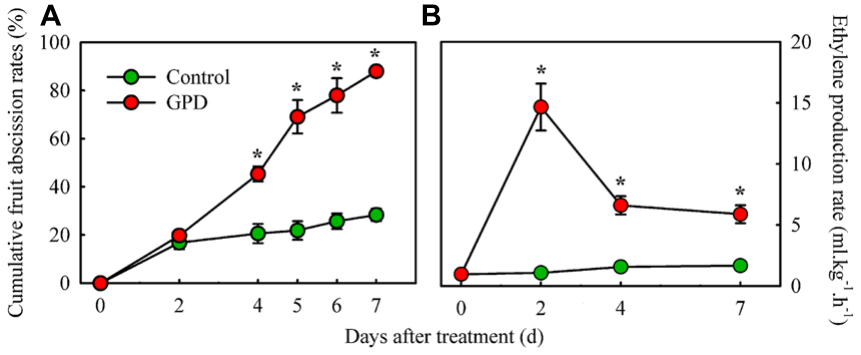
**Effect of the GPD treatment on fruit abscission **(A)** and ethylene production **(B)** in litchi.** Each value represented the means of three biological replicates from three different trees, with the SE indicated by vertical bars. Significant differences at 0.05 level are indicated with asterisk (^∗^) according to *t*-test.

Cumulative fruit abscission rate and ethylene production in fruit were compared between the GPD treatment and the CK (**Figure 1**). The CFARs showed similar trends (**Figure 1A**), which gradually increased during the first 2 days and had no visible difference. Four days after treatment, the CFAR in GPD-treated fruit was significantly higher than that in the CK. Consequently, 88.0% of the fruit had dropped by 7 days after the GPD treatment, in contrast to the 28.0% loss in the CK, indicating that GPD treatment significantly accelerated fruit drop. In addition to the induction of fruit drop, a clear impact on ethylene production was also observed in GPD treated fruit. Within the 7 days of observation, ethylene production of CK fruit remained at a low level below 2 μl⋅kg^-1^⋅h^-1^. In contrast, ethylene production of GPD treated fruit increased rapidly and became 15-fold higher at days 2, then decreased slightly but remained higher level than the CK (sixfolds higher than the CK) until 7 days after treatment (**Figure 1B**). The increase in ethylene production suggested that the GPD treatment probably accelerated fruit drop through the induction of fruit ethylene production.

**FIGURE 2:**
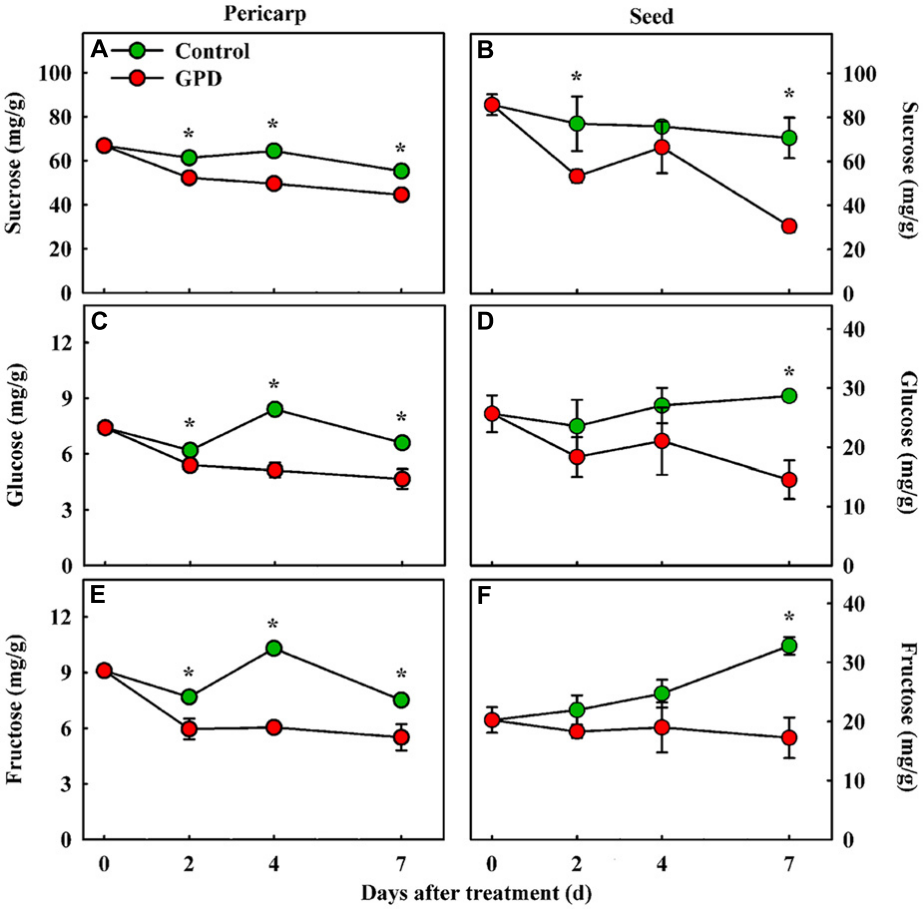
**Change in soluble sugar contents (sucrose, glucose, and fructose) in the fruit pericarp **(A,C,E)** and seed **(B,D,F)** of litchi after the GPD treatment.** Each value represented the means of three biological replicates from three different trees, with the SE indicated by vertical bars. Significant differences at 0.05 level are indicated with asterisk (^∗^) according to *t*-test.

Girdling plus defoliation treatment also led to obvious changes in soluble sugar contents in fruit. The contents of sucrose, glucose, and fructose were determined in both the pericarp and seed tissues. The sucrose content in the GPD treated fruit decreased more quickly, and therefore was significantly lower than those in the CK from 2 to 7 days after treatment in both tissues (**Figures 2A,B**). Although the glucose and fructose concentrations showed a similar change pattern, both of them in CK were significantly higher than those in GPD treated fruit from 2 to 7 days in pericarp (**Figures 2C,E**), and at 7 days in seed (**Figures 2D,F**). These results indicated that blocking carbon nutrient supply to fruit by the GPD treatment caused decrease in soluble sugar contents in fruit, generating carbohydrate stress which might aggravate the fruit abscission.

### Digital Transcript Abundance Profile Analysis and Clustering of Differentially Expressed Genes

To explore the transcriptional changes of litchi fruit in response to carbohydrate stress induced by the GPD treatment, six DTA tag profile libraries of the CK and GPD treated tissue samples (CK2, CK4, CK7, GPD2, GPD4, and GPD7) were constructed and sequenced (**Table 2**). After quality filtering, a total of 34 million reads were generated from the above six libraries (5–6 million reads for each library). The tag sequences of the six libraries were mapped to the litchi fruit transcriptome assembled by Trinity, and 81–89% of all clean reads were matched (**Table 2**), suggesting that we obtained a good quality of sequencing DTA libraries.

**Table 2:** Statistics of DTA libraries.

	Total reads	Total mapped reads^∗^	Unique match reads
CK2	6,053,801	5,092,795 (84.13%)	4,258,546 (70.34%)
CK4	5,064,253	4,120,052 (81.36%)	3,450,681 (68.14%)
CK7	5,225,695	4,253,551 (81.40%)	3,599,995 (68.89%)
GPD2	5,897,341	5,032,315 (85.33%)	4,373,685 (74.16%)
GPD4	5,898,762	5,255,458 (89.09%)	4,655,256 (78.92%)
GPD7	5,898,257	5,047,765 (85.58%)	4,483,144 (76.01%)

*^∗^Number and percentage of reads mapped onto litchi fruit transcriptome assembled by Trinity.*

After comparing the three paired-libraries (CK2 vs. GPD2, CK4 vs. GPD4, and CK7 vs. GPD7), a total of 2,771 DEGs were identified (Supplementary Table S3). Among which, 1,110, 1,368, and 866 DEGs were down-regulated and 180, 92, and 80 were up-regulated in GPD2/CK2, GPD4/CK4, and GPD7/CK7, respectively (Supplementary Figure S3A). GPD2/CK2 and GPD4/CK4 had more DEGs than GPD7/CK7, suggesting that more genes were differentially regulated at the early and middle stages (2 and 4 days) of carbohydrate stress treatment. Venn diagram analysis and a hierarchical clustering showed significant differences in the gene expression profiles between GPD2/CK2 and GPD4/CK4 or between GPD4/CK4 and GPD7/CK7, in contrast to the relatively high similarity between GPD2/CK2 and GPD7/CK7 (Supplementary Figures S3B,C). Noteworthy, a total of 162 DEGs (27 and 135 genes were up- and down-regulated, respectively) were shared in the three comparisons and may represent typical GPD responsive genes.

**FIGURE 3:**
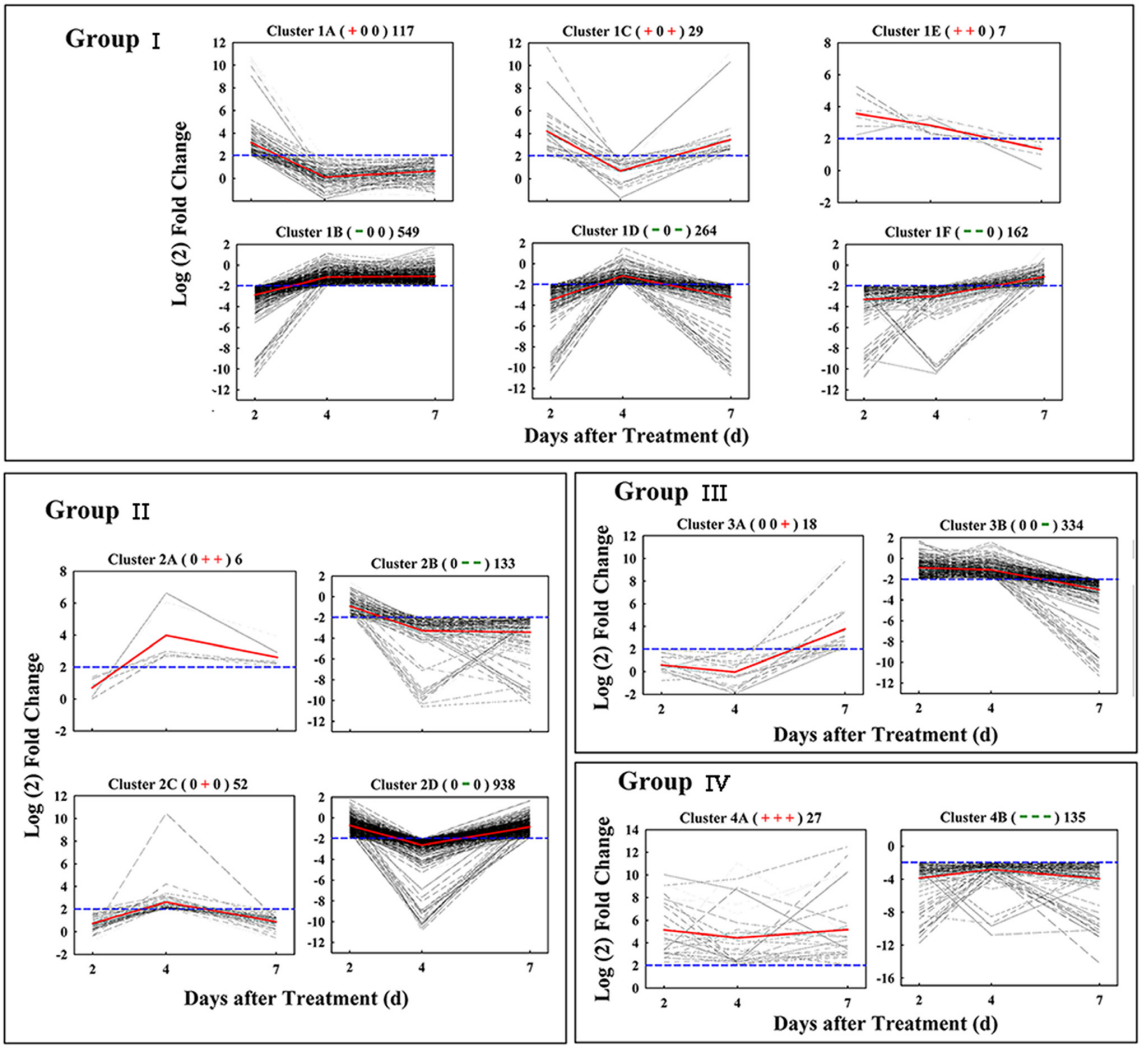
**Gene expression pattern obtained by kinetics-based clustering.** Group I, cluster of DEGs with early and transient changes after GPD-treatment; Group II, clusters of genes modified in their expression until 4 days after GPD-treatment; Group III, cluster of genes with expression kinetics exhibiting late changes after GPD-treatment; Group IV, cluster of DEGs with persistent changes during the whole abscission process. The + and – signs in bracket represent up- and down-regulated of genes, respectively, while 0 represents no change. The numbers on the right of bracket indicate the total numbers of DEGs in each cluster. All of these changes were based on a fourfold change criterion (log2 ratio) indicated in the blue dotted lines. Average values of gene-expression level in clusters were showed in the red solid lines.

Based on similar kinetic patterns of expression, all 2,771 DEGs were classified into 14 types of clusters. They could be further divided into four groups based on their temporal pattern of expression (**Figure 3**). Group I included 1,128 early-responsive genes whose expression were up- or down-regulated early at 2 days after treatment; Group II had 1,129 middle-responsive genes whose expression were not induced or suppressed until 4 days after treatment; Group III contained 353 late-responsive genes that were not regulated until 7 days after treatment; Group IV consisted of 162 constant-responsive genes that up- or down-regulated early and whose expression was maintained constant during the treatment. The genes in Group I and Group IV were considered to function in the early stage of fruit abscission, while Group II and Group III were regulators of the late stage.

### Analysis of the Candidate Genes Involved in Fruit Abscission

According to the results of GO and KEGG enrichment analysis, 907 and 1,124 DEGs were identified in GO (FDR ≤ 0.05) and KEGG pathway (*Q* value ≤ 0.05) with significant enrichment, respectively, (Supplementary Table S4). After eliminating duplicated genes, 857 of the 2,771 DEGs were identified as the candidate genes involved in fruit abscission process induced by the GPD treatment. The up- and down-regulated genes accounted for 13.6 and 86.7%, respectively. According to function annotation, these candidate genes significantly affected by the GPD treatment were divided into 17 functional categories (**Table 3**; Supplementary Table S5).

**Table 3:** Functional categorization of GPD-responsive DEGs.

Functional categories	Genes number	Regulation	Group				Description
			I	II	III	IV	
Carbohydrate metabolism	77	Up	13	2	0	5	Sugar degradation, glycolysis, gluconeogenesis
		Down	18	23	13	3	Sucrose synthesis, glycolysis, transferase, sugar degradation
Chloroplast/photosynthesis	18	Up	0	8	0	0	PSI/II activity, cytochrome b complex
		Down	2	6	2	0	Carbon fixation
Energy/mitochondria	16	Up	0	10	0	0	Electron transport chain
		Down	3	2	1	0	Alternative oxidase
Hormone response	71	Up	5	0	1	1	Ethylene, gibberellin
		Down	36	10	13	5	Abscisic acid, auxin, brassinosteroids, cytokinin, Jasmonic acid
Cell wall modification	144	Up	9	0	1	3	Degradation,
		Down	66	37	18	10	Biosynthesis, degradation, loosening
Transcription factor (TF)	30	Up	2	0	0	0	HEC, LBD
		Down	12	10	5	1	AP2/ERF, bHLH, LBD, GRAS
Signal transduction	78	Up	1	0	0	0	LRR
		Down	29	31	7	10	LRR, CLV, PERK, PI4KB
Cytoskeleton/intracellular transport	40	Up	0	1	0	0	Proton
		Down	9	24	3	3	ABC transporter, microtubule, proton
Cell cycle	6	Up	0	0	0	0	–
		Down	3	2	1	0	Cell division, cyclin
Apoptosis/proteolysis	15	Up	0	0	0	1	–
		Down	5	6	3	0	Protein ubiquitination, protein degradation
Oxidation/reduction	52	Up	7	2	0	1	POD
		Down	26	10	4	2	Rboh, POD, LAC, AO, Rboh
DNA/RNA/protein	39	Up	1	0	0	0	
		Down	7	25	4	2	
Stress/pathogenesis	49	Up	9	2	2	2	Chitinase, PR gene
		Down	17	14	3	0	R gene, lectin
Amino acid metabolism	32	Up	7	0	0	0	Asparagine synthetase, cysteine synthase
		Down	13	9	3	0	Amino acid metabolic process
Lipid metabolism	48	Up	2	0	0	0	–
		Down	23	21	2	0	Lipid/fatty acid synthesis and catabolism
Secondary metabolism	112	Up	11	0	3	1	Alkaloid, flavonoid
		Down	53	24	15	5	Flavonoid, phenolics, phenylpropanoid, terpenoid, vitamin
Others	30	Up	2	0	1	2	–
		Down	11	7	6	1	Cytochrome P450

*A total of 857 GPD-responsive DEGs were classified into 17 functional categories.*

#### Genes Involved in Carbohydrate Metabolism

Seventy-seven carbohydrate metabolism genes were found to be associated with glycolysis, starch, and sucrose degradation, transferase, gluconeogenesis. There were 57 genes expression down-regulated, and among them, 18, 23, 13, and 3 genes belonged to Group I, Group II, Group III, and Group IV, respectively. Of these, many are involved in glycolysis and starch synthesis such as *pyruvate dehydrogenase*, *ATP-citrate synthase*, *alcohol dehydrogenase,* and *sucrose phosphate synthase* (*SPS*), as well as various classes of glycosidase and transferase. In addition, three genes encoding trehalase-6-phosphate phosphatase and trehalose phosphate synthase are also included. In contrast, only 20 carbohydrate metabolism genes were up-regulated. These results indicated that carbohydrate metabolism and sugar signaling pathway were largely inhibited during the entire GPD treatment.

#### Photosynthesis and Energy/Mitochondria Related Genes

We found 18 photosynthesis and 16 energy/mitochondria related genes, 10 and 6 of them were down-regulated, respectively, while the remaining genes were up-regulated. Over 70% genes belonged to Group II, indicating that most genes of these two groups were largely affected at 4 days after the GPD treatment. The expression of genes related to carbon fixation and alternative oxidase was repressed, while the expression of genes associated with PSI and PSII activities, cytochrome b complex and electron transport chain of mitochondria was induced.

#### Plant Hormone Pathway

Seventy one genes were found to be related to plant hormone synthesis and signaling pathways, including those related to auxin (IAA, 17 genes), abscisic acid (ABA, 14 genes), ethylene (13 genes), cytokinin (10 genes), brassinosteroid (BR, 8 genes), gibberellin (GA, 5 genes), and jasmonic acid (JA, 4 genes). Among them, 41, 10, 14, and 6 genes belonged to Group I, Group II, Group III, and Group IV, respectively. Five *1-aminocyclopropane-1-carboxylate oxidase* (*ACO*) genes and another two genes encoding carboxylesterase were induced during the early fruit abscission process after the GPD treatment, and the other six genes were down-regulated. Taking all 17 IAA-related genes as an example, six out of eight genes encoding auxin induced proteins (*Aux/IAA*) and two genes encoding indole-3-acetic acid-amido synthetase (*GH3*) were immediately down-regulated at 2 days after the GPD treatment. Five genes encoding *ARFs* were also repressed at 2 days or 4 days after the GPD treatment. Two genes related to polar auxin transport (*auxin influx carrier protein*) were affected at 7 days after the GPD treatment. These results indicated that most plant hormone related genes were down-regulated at the early stage of the GPD treatment.

#### Responses of Cell Wall Modification

One-hundred and forty four candidate genes, which formed the largest functional category affected by the GPD treatment in our study, were found to be associated with cell wall biosynthesis, loosening, degradation, and modification. Among them, not only all transcripts related to cell wall biosynthesis such as cellulose synthase, but also numerous cell wall degradation and loosening related genes like extensin and pectinesterase were found to be down-regulated. There were only 12 genes associated with cell wall degradation including β-1,3-glucanase, β-D-xylosidase, endoglucanase, xyloglucan endotransglucosylase/hydrolase, pectinesterase, polygalacturonase, and β-D-xylosidase were induced.

#### Expression of Transcription Factor Genes

A total of 30 candidate genes were identified as TFs, including *AP2/ERF*, *bHLH*, *GRAS*, *LOB* domain protein, et cetera. Of them, 14, 10, 5, and 1 genes belonged to Group I, Group II, Group III, and Group IV, respectively, indicating most genes were largely affected at 2 days after the GPD treatment. Apart from *LOB* domain-containing protein and TF *HEC1* in Group I, the remaining 28 genes were down-regulated, suggesting that the vast majority of TFs displayed a repressed expression.

#### Genes Involved in Signal Transduction and Membrane/Cytoskeleton/Intracellular Transport

Seventy-eight GPD-responsive DEGs were found to be associated with signal transduction. Except one gene encoding serine/threonine-protein kinase, the expression of all other 77 genes was repressed. Among them, 29, 31, 7, and 10 genes belonged to Group I, Group II, Group III, and Group IV, respectively, indicating most genes were at low level of expression at 2 and 4 days after the GPD treatment. Of these, many were encoding for LRR receptor-like serine/threonine-protein kinase, receptor protein kinase CLAVATA1 and proline-rich receptor-like protein kinase. On the other hand, 40 genes were found to be involved in cytoskeleton or intracellular transport. Among them, all genes were repressed except one gene encoding for H^+^-transporting two-sector ATPase. These repressed genes included 22 ATP-bind cassette transporters (ABC transporters) and a class of genes related to cytoskeleton function, including those encoding CLIP-associated proteins, formin-like proteins and microtubule-associated proteins.

#### Impacts on Cell Cycle and Proteolysis

Our results showed that only six genes were found to be associated with cell cycle, including two cyclin D genes (Group I), one cyclin-dependent kinase inhibitor gene (Group III), and three cell division related genes (Group I and II). They were largely repressed by the GPD treatment. Moreover, 15 genes involved in proteolysis were also found to be down-regulated. These genes encoded F-box proteins, U-box proteins and other members of the ubiquitin ligase complex in potential ubiquitylation pathway, like E3 ubiquitin-protein ligase and the RING-H2 finger protein. A few genes related to protein hydrolysis, such as subtilisin-like protease and serine carboxypeptidase, were repressed as well.

#### Responses of Stress/Pathogenesis and Oxidation/Reduction

There were 49 genes involved in stress/pathogenesis response pathway. Among them, 26, 16, 5, and 2 genes belonged to Group I, Group II, Group III, and Group IV, respectively, indicating most genes showed great difference in expression at the early stage. Of them, 11 genes exhibited abrupt up-regulated expression at 2 days, including those encoding chitinase, pathogenesis-related proteins, and wound-induced proteins. Whereas, most of the other remaining genes were down-regulation at early or late fruit abscission.

Fifty-two genes were found to be involved in oxidation/reduction processes. Among them, 33, 12, 4, and 3 genes belonged to Group I, Group II, Group III, and Group IV, respectively. Of 10 up-regulated genes, seven genes showed increased transcript abundance at 2 days after the GPD treatment, including those encoding peroxidase, inositol oxygenase, and thioredoxin. Forty-two genes with inhibited expression included genes encoded for laccases (13 genes), peroxidases (10 genes), respiratory burst oxidase homolog proteins (three genes), glutaredoxins (four genes), and L-ascorbate oxidases (three genes).

### Validation of the Candidate Genes by qRT-PCR

**FIGURE 4:**
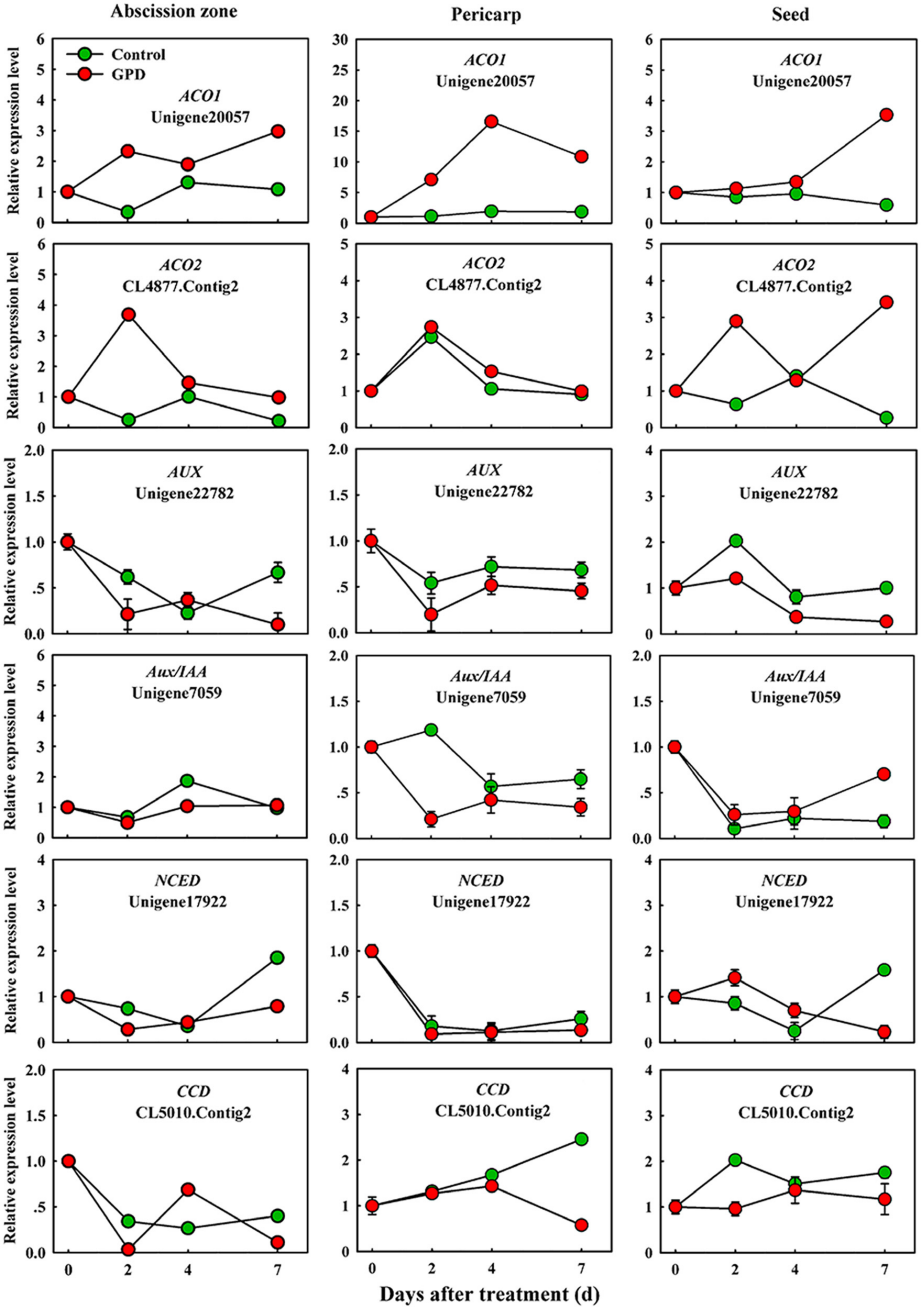
**Real-time quantitative PCR analysis of the expression of GPD-responsive DEGs related to hormone signaling and metabolism.** The results are means of three biological replicates (±SE). The value of transcript levels in CK was arbitrarily set to 1.

**FIGURE 5:**
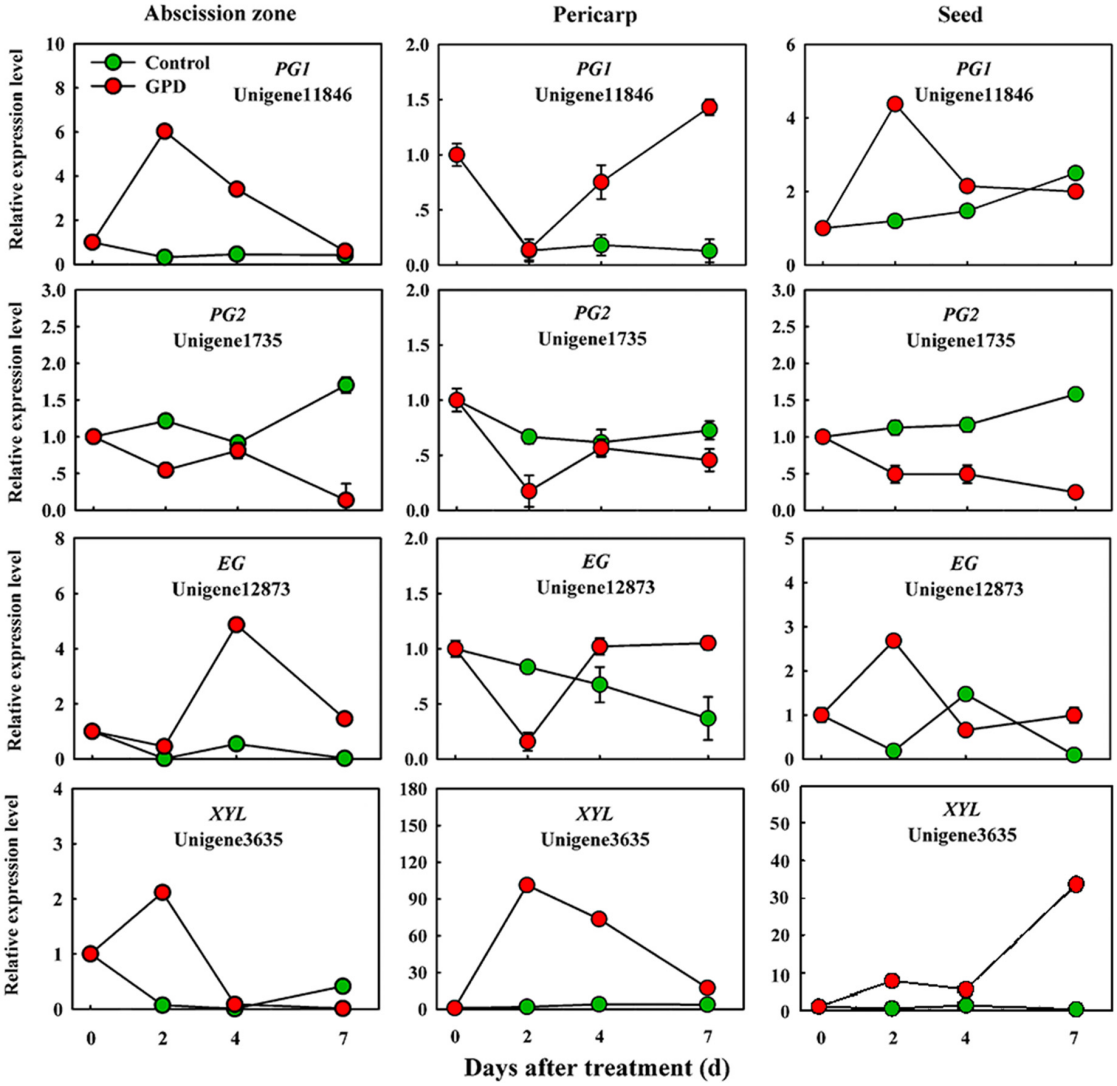
**Real-time quantitative PCR analysis of the expression of GPD-responsive DEGs related to cell wall degradation.** The results are means of three biological replicates (±SE). The value of transcript levels in CK was arbitrarily set to 1.

**FIGURE 6:**
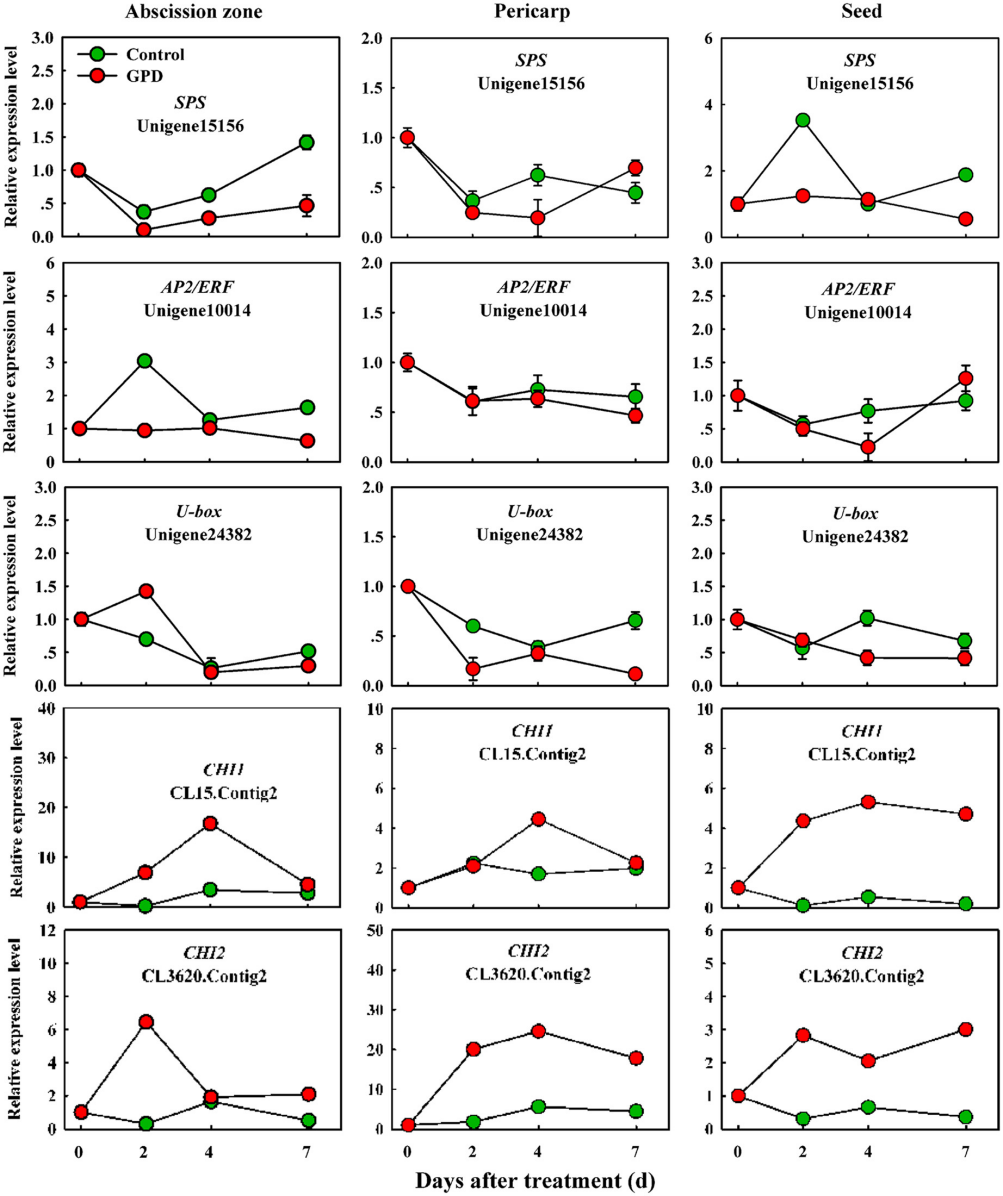
**Real-time quantitative PCR analysis of the expression of GPD-responsive DEGs related to carbohydrate metabolism, transcription factor (TF) activities, potential ubiquitylation and stress response.** The results are means of three biological replicates (±SE). The value of transcript levels in CK was arbitrarily set to 1.

In order to verify the expression pattern of those genes involved in fruit abscission, seven up-regulated and eight down-regulated genes were selected for further qRT-PCR analysis. These genes belonged to divergent functional categories or pathways. Six genes (*ACO1*, *ACO2*, *AUX*, *Aux/IAA*, *NCED,* and *CCD*) were implicated in plant hormones (ethylene, auxin, and abscisic acid) regulation pathway, four genes (*PG1*, *PG2*, *EG,* and *XYL*) were involved in cell wall modification, the remaining five genes (*SPS*, *AP2/ERF*, *U-box,* and *CHI1*, *CHI2*) were assigned to carbohydrate metabolism, TFs, potential ubiquitylation and stress response. **Figure 4** showed that the expressions of *ACO1* and *ACO2* were increased and *AUX* and *CCD* transcript abundance were decreased in three tissues (AZ, pericarp and seed) at 2 and 7 days after the GPD treatment; the expressions of *NCED* in AZ and *AUX/IAA* in pericarp were also depressed. The transcript mRNAr level of *PG1*, *EG*, *XYL*, *CHI1,* and *CHI2* were increased and *PG2*, *SPS*, *AP2/ERF,* and *U-box* were inhibited in all the tissues (**Figures 5** and **6**). Although the exact fold changes of the selected genes at several data points varied between DTA profile and qRT-PCR analysis, their expression trends found from the two different approaches were similar. These results re-confirmed the accuracy of our DTA profile data and indicated that AZ was the more important tissue than pericarp and seed during the fruit abscission caused by the GPD treatment in litchi.

## Discussion

Fruit is a heterotrophic organ dependent mainly on the supply of photosynthetic products from leaves. A strong connection between the carbohydrate availability for fruit and their probability of abscission has been described in citrus (Ruiz et al., 2001; Iglesias et al., 2006), apple (Zhu et al., 2011), and also litchi (Yuan and Huang, 1988). In this study, almost 90% of the fruits on branches dropped after treated by GPD for 7 days, about three times higher than the CK. In the meantime, GPD also significantly decreased the content of sugars and increased ethylene production in fruit. GPD completely blocks the supply of carbohydrate to fruit, which has been proved to be reliable and repeatable experimental model for the research of fruit abscission under carbohydrate stress in litchi (Kuang et al., 2012; Peng et al., 2013). Our previous studies used this experimental model to verify few cloned genes related to auxin signaling (Kuang et al., 2012), cell wall degradation (Peng et al., 2013) and ethylene biosynthesis (Wu et al., 2013) involved in litchi fruitlet drop. Our present work aimed at identification of molecular components involved in fruit abscission in litchi, an important fruit crop, for which reference genome is not available now. Thus, this study provided the first report of gene expression profile related to fruit abscission in response to carbohydrate stress on the whole transcriptome level in litchi.

RNA-Seq has been successfully used to sequence the transcriptome of many non-model organisms. *De novo* transcriptome assembly from short reads is improving with the development of advanced bioinformatics softwares (Schulz et al., 2012). In this study, four publicly available assemblers were used to evaluate the litchi fruit RNA-Seq data generated previously (Li et al., 2013). Trinity resulted in a better *de novo* transcriptome assembly compared to other software tested, while previously used SOAPdenovo performed the worst due to its lowest utilized reads. Similar results were reported by Garg et al. (2011) and Zhao et al. (2011). It is not surprising, but having a better assembly for litchi transcriptome is worthwhile and highly desirable. A total of 2,771 significantly DEGs were screened as GPD-responsive genes and 857 of them were identified as the candidate genes involved in fruit abscission process. In general, abscission is considered to develop through four major steps: the AZ differentiation, the competence to abscission signals, the activation of abscission, and the differentiation of a protective layer (Estornell et al., 2013). In litchi, differentiation of fruit AZ may have occurred very early during flower differentiation (unpublished data). And the second abscission step may occur 0–2 days after the GPD treatment, which might be considered as the early phase of GPD-induced fruit abscission. In this phase, CFAR in the GPD treatment was slowly increased and almost same as the CK, but ethylene release was rapidly risen and significantly higher than the CK. Meanwhile, the expression of 403 and 58 genes belonging to Group I and Group IV, respectively, were changed, which probably leads to acquisition of abscission sensitivity and competence. Therefore, all these genes related to carbohydrate metabolism, hormone response, TFs, and kinase activities, could be directly response to GPD and play an important role on fruit abscission. From 2 to 7 days after treatment, the CFAR in the GPD treatment was largely aggravated at an increasing rate and significantly higher than that in the CK, indicating fruit abscission was being activated and executed. It might be regarded as the late phase of GPD-induced fruit abscission, involving changes in expression of genes in Group II and Group III. A key step in this stage is cell separation, which is mostly induced by cell wall degrading enzymes such as polygalacturonase.

With carbohydrate supply being cut off, it is not surprising that a sharp decline in the soluble sugars level in pericarp and seed occurred after GPD treatment. A reduction of sugar concentration was already reported as a reaction to sugar starvation in citrus (Gómez-Cadenas et al., 2000; Iglesias et al., 2006). In the early phase of the abscission process, we observed a high expression level of genes involved in starch and sucrose degradation, glycolysis and gluconeogenesis, and a decrease in the expression of genes for sucrose synthesis, such as *SPS*. While in the late phase of the abscission process, a high expression level of genes related to energy metabolism and PSI/II activity and a low expression level of genes associated with carbon fixation and carbohydrate metabolism occurred. These results implicated the reduction in sink storage of GPD-treated fruit and the energy consumption for maintenance. The high expression of carbohydrate metabolism genes was also found in apple (Zhu et al., 2011) and litchi (Li et al., 2013) after shading treatment, while the photosynthesis related genes were inhibited. However, sorbitol metabolism seemed to be more important in apple fruitlet drop (Zhu et al., 2011), which was not found in litchi.

Although the relationships among the hormonal signals, carbohydrate shortage and abscission are still not clearly elucidated, it is widely believed that the endogenous balance of ethylene and auxin in the AZ affects organ abscission. In this study, ethylene production increased and peaked in GPD-treated fruit, coinciding with the up-regulation of genes encoding ethylene biosynthesis (*ACO1* and *ACO2*), and it happened prior to massive fruit drop. On the contrary, auxin responsive, and transport related genes, such as *Aux/IAA* and *AUX*, were all repressed by GPD. These results agreed with a previous report, where the GPD treatment consistently reduced endogenous auxin content and altered auxin responsive genes in litchi (Kuang et al., 2012). It indicated that auxin signaling and influx were impaired prior to the onset of fruit drop. These results were consistent with other studies on abscission, like mature fruit abscission in melon (Corbacho et al., 2013) and olive (Gil-Amado and Gomez-Jimenez, 2013), young fruit drop induced by NAA and shading in apple (Zhu et al., 2011). It has been proposed that ABA might be implicated in response to nutrient stress (Yang et al., 2003), and correlate with the activation of ethylene-associated abscission in citrus fruitlet (Gómez-Cadenas et al., 2000), shading-induced abscission of apple fruitlet (Zhu et al., 2011), and mature abscission in melon fruit (Corbacho et al., 2013). However, we observed a low expression of genes involved in ABA biosynthesis and signaling during the early and/or late stages of fruit abscission in response to the GPD treatment, suggesting that ABA might be involved in litchi fruit abscission through another pathway, rather than integrating with ethylene pathway, as shown in apple (Zhu et al., 2011). In addition, our study indicated that besides the participation of ethylene and auxin in controlling abscission events, other hormones, such as ABA, BR, JA, GA, and cytokinin apparently participated in an intricate interaction network regulating the abscission of GPD-treated fruits.

In *Arabidopsis* floral abscission (Burr et al., 2011) and melon fruit abscission (Corbacho et al., 2013), LRR receptor-like protein kinase (LRR-RLKs) has been thought to be a potential controller by competing activities. Here, expression analysis detected many LRR-RLKs down-regulated during the whole fruit abscission process induced by the GPD treatment, suggesting the involvement of LRR-RLKs in regulating litchi fruit abscission. In addition, a subset of genes involved in microtubule depolymerization and a group of gene encoding ABC transporter showed low abundance at 4–7 days after the GPD treatment. These results indicated that changes in integrity of cytoskeleton and the intracellular transport system might be involved in the late stage of litchi fruit abscission. On the other hand, late events were potentially controlled by down-regulation of *AP2/ERF* and *GRAS* TFs, while the early events may be controlled by down-regulation of *AP2/ERF*, *bHLH,* and *LOB* domain proteins.

The last key step in promoting AZ cell separation that eventually result in the shedding of organs is the induction of cell wall degrading and modifying enzymes, such as PGs, cellulases, xyloglucan endotransglucosylase/hydrolases (XTHs), and expansins (Singh et al., 2011; Zhu et al., 2011; Corbacho et al., 2013). However, not only numerous transcripts related to cell wall biosynthesis but also cell wall degradation and loosening related genes were found to be down-regulated during GPD-induced abscission process. Several cell wall degradation genes, such as *PG1*, *EG,* and *XYL*, was strongly up-regulated, indicating that these genes might play an essential and positive regulation role in cell separation at the early stage of litchi fruit abscission.

**FIGURE 7:**
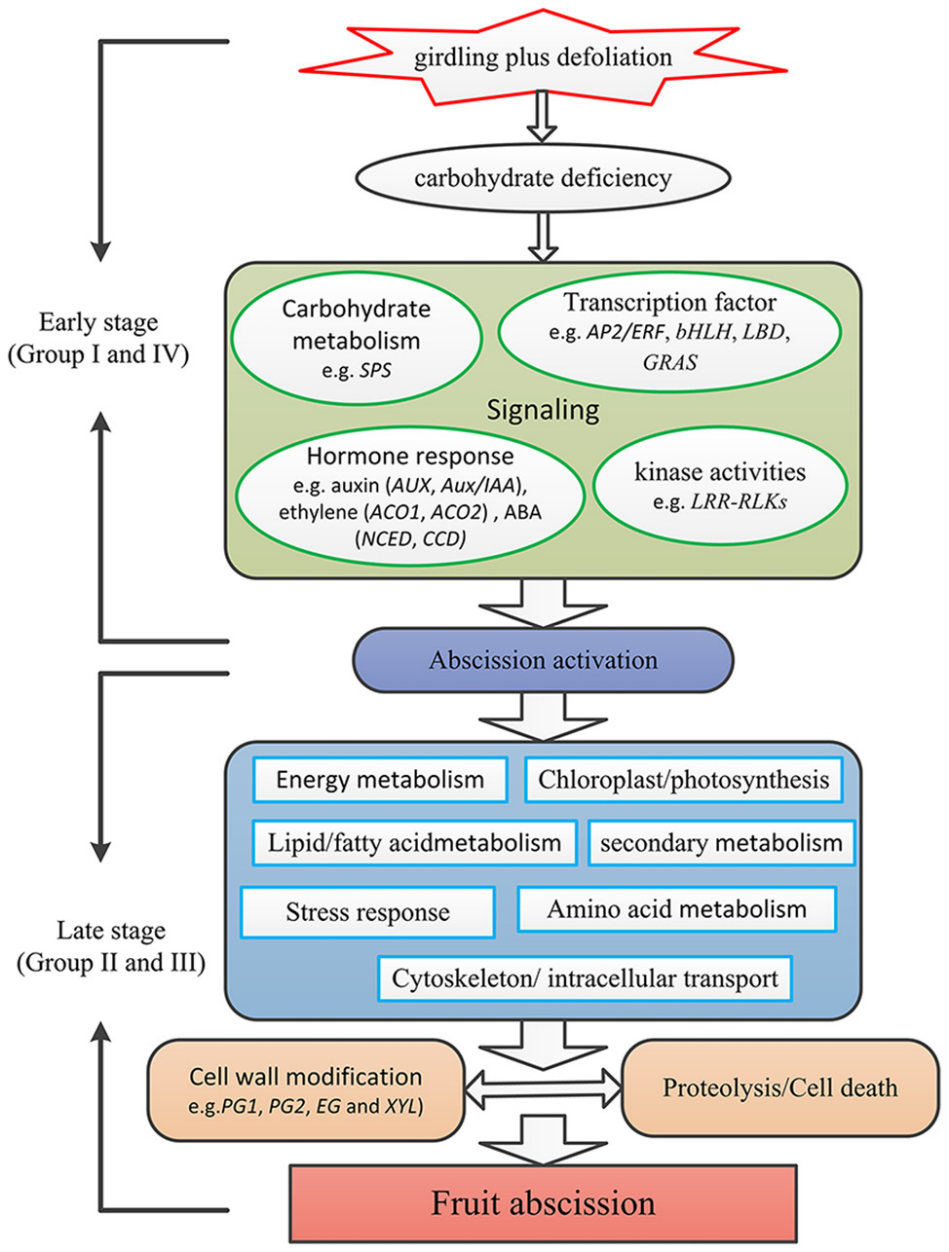
**A preliminary framework of the gene network involved in litchi fruit abscission induced by carbohydrate stress.** Firstly fruit perceive carbohydrate stress induced by the GPD treatment. The sugar starvation signal induces changes of cellular metabolisms, such as hormone signal transduction (e.g., ethylene, auxin, and ABA), protein kinase activities (e.g., *LRR-RLKs*), and TF activities (e.g., *AP2/ERF*, *bHLH*, *LBD*, *GRAS*). Other metabolism pathways involving integrity of cytoskeleton, smoothness of intracellular transport, lipid/fatty acid metabolism, amino acid metabolism as well as secondary metabolism also take place during the process. Finally, protein hydrolysis, cell separation and cell death occur, leading to the litchi fruit abscission through the breakdown of the balance between cell damage and development.

Based on our results, a preliminary framework of the gene network involved in litchi fruit abscission was proposed (**Figure 7**). Fruit may first perceive carbohydrate stress induced by the GPD treatment. Then, the sugar starvation signal transduction might induce changes of cellular metabolisms, such as hormone signal transduction (e.g., ethylene, auxin, and ABA), protein kinase activities (e.g., *LRR-RLKs*), and TF activities (e.g., *AP2/ERF*, *bHLH*, *LBD*, *GRAS*). Other metabolism pathways could also take place in this process, such as integrity of cytoskeleton, smoothness of intracellular transport, lipid/fatty acid metabolism, amino acid metabolism as well as secondary metabolism. Finally, protein hydrolysis, cell separation, and cell death occur, leading to the litchi fruit abscission through the break of the balance between cell damage and development.

## Conclusion

A litchi fruit transcriptome assembly was greatly improved in this work using Trinity software. 857 GPD-responsive DEGs were identified as candidate genes involved in the process of litchi fruit abscission induced by carbohydrate stress. A hypothetical molecular model for litchi fruit abscission induced by the GPD treatment was proposed based on the results. Our study provided the first information about gene expression profile related to fruit abscission induced by carbohydrate stress on whole transcriptome level, which contributes to a better understanding for the molecular regulatory mechanism of fruit abscission in litchi. Further studies should focus on the functional characterization of genes involved in the above pathways.

## Author Contributions

JL designed the research and finalized the manuscript. CL conceived and drafted this paper. YW performed most of the biological experiments. YW and JL conducted bioinformatics analyses and data interpretation. XH and HW revised the manuscript. All the authors approved the final manuscript.

## Conflict of Interest Statement

The authors declare that the research was conducted in the absence of any commercial or financial relationships that could be construed as a potential conflict of interest.

## Footnotes

1 http://soap.genomics.org.cn

2 http://soap.genomics.org.cn/SOAPdenovo-Trans.html

3 http://www.ebi.ac.uk/~zerbino/velvet/

4 http://www.ebi.ac.uk/~zerbino/oases/

5 http://sourceforge.net/projects/trinityrnaseq

6 http://www.ncbi.nlm.nih.gov

7 http://www.expasy.ch/sprot

8 http://www.genome.jp/kegg

## ABBREVIATIONS

AZ: abscission zone
CFAR: the cumulative fruit abscission rate
CK: control
DEG: differentially expressed gene
DTA: digital transcript abundance
GO: Gene Ontology
GPD: girdling plus defoliation
KEGG: Kyoto encyclopedia of genes and genomes.

## References

[B1] Agustí J. Merelo P. Cercós M. Tadeo F. R. Talón M. (2009). Comparative transcriptional survey between laser-microdissected cells from laminar abscission zone and petiolar cortical tissue during ethylene-promoted abscission in citrus leaves. *BMC Plant Biol.* 9:127 10.1186/1471-2229-9-127 PMC2770498 19852773

[B2] Audic S. Claverie J. M. (1997). The significance of digital gene expression profiles. *Genome Res.* 7 986–995. 10.1101/gr.7.10.986 9331369

[B3] Berüter J. Droz P. (1991). Studies on locating the signal for fruit abscission in the apple tree. *Sci. Hortic.* 46 201–214. 10.1016/0304-4238(91)90043-X

[B4] Botton A. Eccher G. Forcato C. Ferrarini A. Begheldo M. Zermiani M. (2011). Signaling pathways mediating the induction of apple fruitlet abscission. *Plant Physiol.* 155 185–208. 10.1104/pp.110.165779 21037112 PMC3075760

[B5] Burr C. A. Leslie M. E. Orlowski S. K. Chen I. Wright C. E. Daniels M. J. (2011). CAST AWAY, a membrane-associated receptor-like kinase, inhibits organ abscission in *Arabidopsis*. *Plant Physiol.* 156 1837–1850. 10.1104/pp.111.175224 21628627 PMC3149937

[B6] Chang J. C. Lin T. S. (2007). Gas exchange in litchi under controlled and field conditions. *Sci. Hortic.* 114 268–274. 10.1016/j.scienta.2007.06.023

[B7] Chang J. C. Lin T. S. (2008). Fruit yield and quality as related to flushes of bearing shoots in litchi. *J. Am. Soc. Hortic. Sci.* 133 284–289.

[B8] Cho S. K. Larue C. T. Chevalier D. Wang H. Jinn T. L. Zhang S. (2008). Regulation of floral organ abscission in *Arabidopsis thaliana*. *Proc. Natl. Acad. Sci. U.S.A.* 105 15629–19634. 10.1073/pnas.0805539105 18809915 PMC2563077

[B9] Conesa A. Gotz S. Garcia-Gomez J. M. Terol J. Talon M. Robles M. (2005). Blast2GO: a universal tool for annotation, visualization and analysis in functional genomics research. *Bioinformatics* 21 3674–3676. 10.1093/bioinformatics/bti610 16081474

[B10] Corbacho J. Romojaro F. Pech J. C. Latche A. Gomez-Jimenez M. C. (2013). Transcriptomic events involved in melon mature-fruit abscission comprise the sequential induction of cell-wall degrading genes coupled to a stimulation of endo and exocytosis. *PLoS ONE* 8:e58363 10.1371/journal.pone.0058363 PMC3590154 23484021

[B11] Estornell L. H. Agusti J. Merelo P. Talon M. Tadeo F. R. (2013). Elucidating mechanisms underlying organ abscission. *Plant Sci.* 199 48–60. 10.1016/j.plantsci.2012.10.008 23265318

[B12] Garg R. Patel R. K. Tyagi A. K. Jain M. (2011). De novo assembly of chickpea transcriptome using short reads for gene discovery and marker identification. *DNA Res.* 18 53–63. 10.1093/dnares/dsq028 21217129 PMC3041503

[B13] Ghangal R. Chaudhary S. Jain M. Purty R. S. Chand S. P. (2013). Optimization of de novo short read assembly of seabuckthorn (*Hippophae rhamnoides* L.) transcriptome. *PLoS ONE* 8:e72516 10.1371/journal.pone.0072516 PMC3749127 23991119

[B14] Gil-Amado J. A. Gomez-Jimenez M. C. (2013). Transcriptome analysis of mature fruit abscission control in olive. *Plant Cell Physiol.* 54 244–269. 10.1093/pcp/pcs179 23292600

[B15] Gómez-Cadenas A. Mehouachi J. Tadeo F. R. Primo-Millo E. Talon M. (2000). Hormonal regulation of fruitlet abscission induced by carbohydrate shortage in citrus. *Planta* 210 636–643. 10.1007/s004250050054 10787058

[B16] Grabherr M. G. Haas B. J. Yassour M. Levin J. Z. Thompson D. A. Amit I. (2011). Full-length transcriptome assembly from RNA-Seq data without a reference genome. *Nat. Biotechnol.* 29 644–652. 10.1038/nbt.1883 21572440 PMC3571712

[B17] Hieke S. Menzel C. M. Doogan V. J. Ludders P. (2002). The relationship between yield and assimilate supply in lychee (*Litchi chinensis* Sonn.). *J. Hortic. Sci. Biotechnol.* 77 326–332.

[B18] Hu Z. Q. Wang H. C. Hu G. B. (2005). Measurement of sugars, organic acids and vitamin C in litchi fruit by high performance liquid chromatography. *J. Fruit Sci.* 5 34 10.3969/j.issn.1009-9980.2005.05.035

[B19] Iglesias D. J. Tadeo F. R. Primo-Millo E. Talon M. (2006). Carbohydrate and ethylene levels related to fruitlet drop through abscission zone A in citrus. *Trees* 20 348–355. 10.1007/s00468-005-0047-x

[B20] Kuang J. F. Wu J. Y. Zhong H. Y. Li C. Q. Chen J. Y. Lu W. J. (2012). Carbohydrate stress affecting fruitlet abscission and expression of genes related to auxin signal transduction pathway in litchi. *Int. J. Mol. Sci.* 13 16084–16103. 10.3390/ijms131216084 23443112 PMC3546680

[B21] Li C. Q. Wang Y. Huang X. M. Li J. Wang H. C. Li J. G. (2013). De novo assembly and characterization of fruit transcriptome in *Litchi chinensis* Sonn. and analysis of differentially regulated genes in fruit in response to shading. *BMC Genomics* 14:552 10.1186/1471-2164-14-552 PMC3751308 23941440

[B22] Li J. G. Liu S. Z. Wang Z. H. (2004). Changes in endogenous polyamine contents during fruit development of litchi (*Litchi chinensis*). *Plant Physiol. Commun.* 40 153–156.

[B23] Li R. Yu C. Li Y. Lam T. W. Yiu S. M. Kristiansen K. (2009). SOAP2: an improved ultrafast tool for short read alignment. *Bioinformatics* 25 1966–1967. 10.1093/bioinformatics/btp336 19497933

[B24] Mehouachi J. Serna D. Zaragoza S. Agusti M. Talon M. Primo-Millo E. (1995). Defoliation increases fruit abscission and reduces carbohydrate levels in developing fruits and woody tissues of *Citrus unshiu*. *Plant Sci.* 107 189–197. 10.1016/0168-9452(95)04111-7

[B25] Meir S. Philosoph-Hadas S. Sundaresan S. Selvaraj K. S. Burd S. Ophir R. (2010). Microarray analysis of the abscission-related transcriptome in the tomato flowerabscission zone in response to auxin depletion. *Plant Physiol.* 154 1929–1956. 10.1104/pp.110.160697 20947671 PMC2996037

[B26] Mitra S. K. Pereira L. S. Pathak P. K. Majumdar D. (2003). Fruit abscission pattern of lychee cultivars. *Acta Hortic.* 665 215–218.

[B27] Mortazavi A. Williams B. A. McCue K. Schaeffer L. Wold B. (2008). Mapping and quantifying mammalian transcriptomes by RNA-Seq. *Nat. Methods* 5 621–628. 10.1038/nmeth.1226 18516045 PMC13303166

[B28] Moss G. I. Steer B. T. Kriedemann P. E. (1972). The regulatory role of inflorescence leaves in fruit-setting by sweet orange (*Citrus sinensis*). *Physiol. Plant.* 27 432–438. 10.1111/j.1399-3054.1972.tb03639.x

[B29] Peng G. Wu J. Y. Lu W. J. Li J. G. (2013). A polygalacturonase gene clustered into clade E involved in lychee fruitlet abscission. *Sci. Hortic.* 150 244–250. 10.1016/j.scienta.2012.10.029

[B30] Powell A. A. Krezdorn A. H. (1977). Influence of fruit setting treatment on translocation of 14C-metabolites in citrus during flowering and fruiting. *J. Am. Soc. Hortic. Sci.* 102 709–714.

[B31] Ruiz R. Garca-Luis A. Monerri C. Guardiola J. L. (2001). Carbohydrate availability in relation to fruitlet abscission in citrus. *Ann. Bot.* 87 805–812. 10.1006/anbo.2001.1415

[B32] Schulz M. H. Zerbino D. R. Vingron M. Birney E. (2012). Oases: robust de novo RNA-seq assembly across the dynamic range of expression levels. *Bioinformatics* 28 1086–1092. 10.1093/bioinformatics/bts094 22368243 PMC3324515

[B33] Singh A. P. Tripathi S. K. Nath P. Sane A. P. (2011). Petal abscission in rose is associated with the differential expression of two ethylene-responsive xyloglucan endotransglucosylase/hydrolase genes, RbXTH1 and RbXTH2. *J. Exp. Bot.* 62 5091–5103. 10.1093/jxb/err209 21765161 PMC3193013

[B34] Stern R. A. Adato I. Goren M. Eisenstein D. Gazit S. (1993). Effects of autumnal water stress on litchi flowering and yield in Israel. *Sci. Hortic.* 54 295–302. 10.1016/0304-4238(93)90108-3

[B35] Stern R. A. Kigel J. Tomer E. Gazit S. (1995). ‘Mauritius’ lychee fruit development and reduced abscission after treatment with the auxin 2,4,5-TP. *J. Am. Soc. Hortic. Sci.* 120 65–70.

[B36] Talon M. Tadeo F. R. Ben-Cheikh W. Gomez-Cadenas A. Mehouachi J. Pérez-Botella J. (1997). Hormonal regulation of fruit set and abscission in citrus: classical concepts and new evidence. *Acta Hortic.* 463 209–218.

[B37] Trueman S. J. Turnbull C. G. N. (1994). Fruit set, abscission and dry matter accumulation on girdled branches of macadamia. *Ann. Bot.* 74 667–674. 10.1006/anbo.1994.1169

[B38] Wang X. Liu D. M. Li A. L. Sun X. L. Zhang R. Z. Wu L. (2013). Transcriptome analysis of tomato flower pedicel tissues reveals abscission zone-specific modulation of key meristem activity genes. *PLoS ONE* 8:e55238 10.1371/journal.pone.0055238 PMC3563536 23390523

[B39] Wu J. Y. Li C. Q. Lu W. J. Li J. G. (2013). Cloning of Lc-ACO1 and its expression related to fruitlet abscission in litchi. *J. Fruit Sci.* 30 207–213.

[B40] Xiang X. Qiu Y. P. Zhang Z. W. (1995). Endogenous hormones in the fruit of *Litchi chinensis* cv. Nuomici relating to fruit abscission. *J. Fruit Sci.* 12 88–92.

[B41] Xie Y. L. Wu G. X. Tang J. B. Luo R. B. Patterson J. Liu S. L. (2014). SOAPdenovo-Trans: de novo transcriptome assembly with short RNA-Seq reads. *Bioinformatics* 30 1660–1666. 10.1093/bioinformatics/btu077 24532719

[B42] Yan S. C. Chen J. Y. Yu W. M. Kuang J. F. Chen W. X. Li X. P. (2011). Expression of genes associated with ethylene-signalling pathway in harvested banana fruit in response to temperature and 1-MCP treatment. *J. Sci. Food Agric.* 91 650–657. 10.1002/jsfa.4226 21302318

[B43] Yang J. C. Zhang J. H. Wang Z. Q. Zhu Q. S. Liu L. J. (2003). Involvement of abscisic acid and cytokinins in the senescence and remobilization of carbon reserves in wheat subjected to water stress during grain filling. *Plant Cell Environ.* 26 1621–1631. 10.1046/j.1365-3040.2003.01081.x 12172848

[B44] Yuan R. C. Huang H. B. (1988). Litchi fruit abscission: its patterns, effect of shading and relation to endogenous abscisic acid. *Sci. Hortic.* 36 281–292. 10.1016/0304-4238(88)90063-5

[B45] Yuan R. C. Huang H. B. (1992). Improvement of fruit-set in *Litchi chinensis* Sonn. through regulation of source-sink relationships. *J. S. China Agric. Univ.* 13 136–141.

[B46] Yuan R. C. Huang H. B. (1993). Regulation of root and shoot growth and fruit drop of young litchi trees by trunk girdling in view of source-sink relationship. *J. Fruit Sci.* 10 195–198.

[B47] Yuan W. Q. Huang X. M. Wang H. C. Li J. G. Chen H. B. Yin J. H. (2009). Seasonal changes in carbon nutrition reserve in Nuomici *Litchi* trees and its relation to fruit load. *Acta Hortic. Sinica* 35 1533–1538.

[B48] Zerbino D. R. Birney E. (2008). Velvet: algorithms for de novo short read assembly using de Bruijn graphs. *Genome Res.* 18 821–829. 10.1101/gr.074492.107 18349386 PMC2336801

[B49] Zhang J. Z. Zhao K. Ai X. Y. Hu C. G. (2014). Involvements of PCD and changes in gene expression profile during self-pruning of spring shoots in sweet orange (*Citrus sinensis*). *BMC Genomics* 15:892 10.1186/1471-2164-15-892 PMC4209071 25308090

[B50] Zhao Q. Y. Wang Y. Kong Y. M. Luo D. Li X. Hao P. (2011). Optimizing de novo transcriptome assembly from short-read RNA-Seq data: a comparative study. *BMC Bioinformatics* 12(Suppl. 14):S2 10.1186/1471-2105-12-S14-S2 PMC3287467 22373417

[B51] Zhou X. J. Yan H. D. Bai H. H. Yao W. D. (1999). Carbohydrate and endohormone status in relation to fruit set as influenced by trunk spiral girdling of young litchi trees. *Acta Hortic. Sinica* 26 77–80. 10.3321/j.issn:0513-353X.1999.02.002

[B52] Zhu H. Dardick C. D. Beers E. P. Callanhan A. M. Xia R. Yuan R. C. (2011). Transcriptomics of shading-induced and NAA-induced abscission in apple (*Malus domestica*) reveals a shared pathway involving reduced photosynthesis, alterations in carbohydrate transport and signaling and hormone crosstalk. *BMC Plant Biol.* 11:138 10.1186/1471-2229-11-138 PMC3217944 22003957

