# Supplementary figures and images for: An improved fruit transcriptome and the identification of the candidate genes involved in fruit abscission induced by carbohydrate stress in litchi
Source: Front Plant Sci. 2015 Jun 15;6:439. doi: 10.3389/fpls.2015.00439 (PMC4466451; doi:10.3389/fpls.2015.00439)

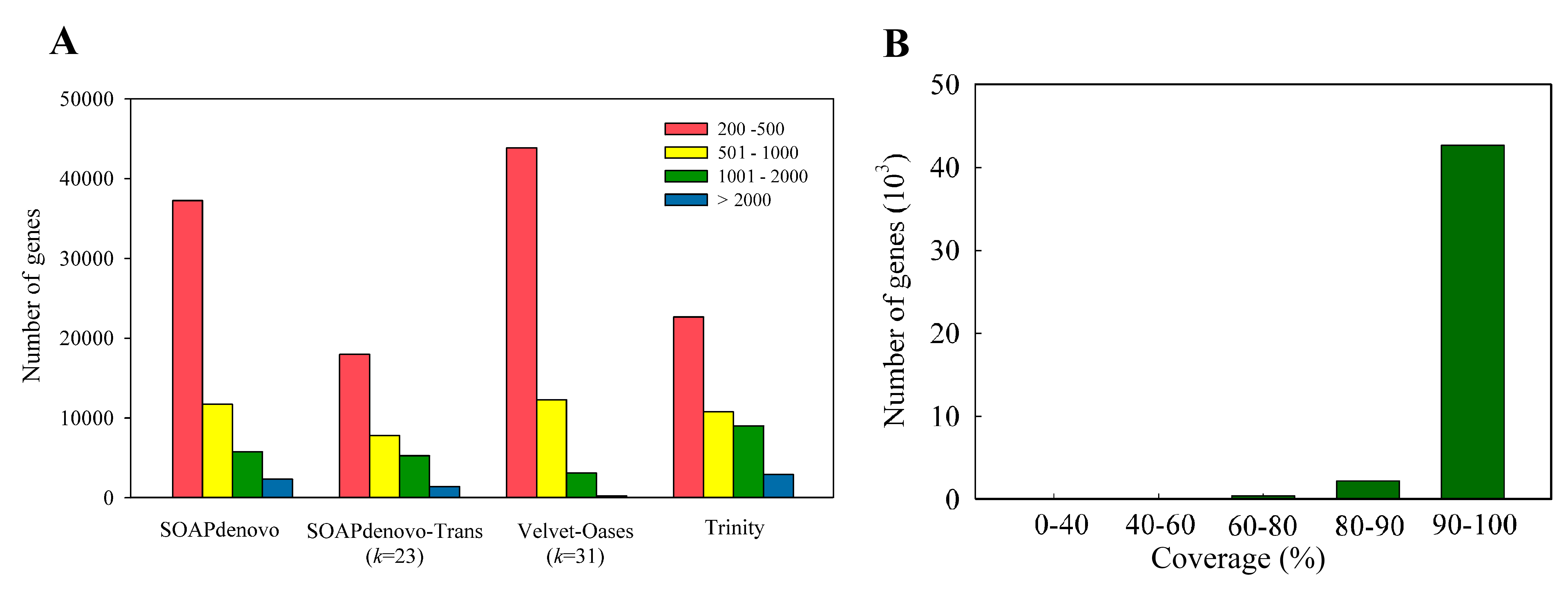

Supplement: Supplementary file 7 [file Image_2.TIF]

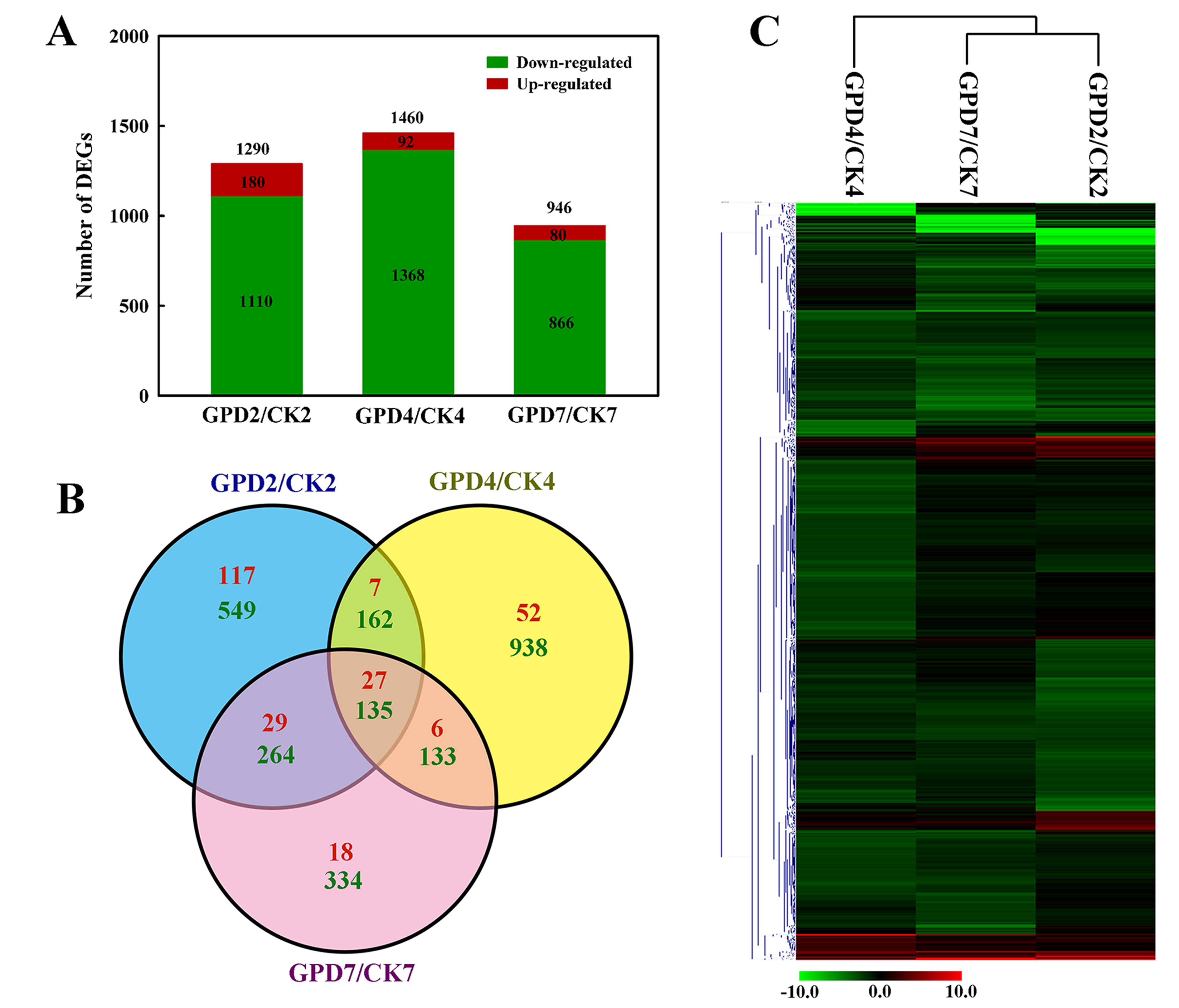

Supplement: Supplementary file 8 [file Image_3.TIF]
